# Omics in Keratoconus: From Molecular to Clinical Practice

**DOI:** 10.3390/jcm14072459

**Published:** 2025-04-03

**Authors:** Sandra Carolina Durán-Cristiano, Andres Bustamante-Arias, Geysson Javier Fernandez, Alba Martin-Gil, Gonzalo Carracedo

**Affiliations:** 1 Grupo de Investigación en Ciencias Básicas, Facultad de Medicina, Universidad CES, Medellín 050010, Colombia; 2 Ocupharm Research Group, Universidad Complutense de Madrid, 28007 Madrid, Spain; amarting@ucm.es (A.M.-G.); jgcarrac@ucm.es (G.C.); 3 Clínica de Oftalmología Sandiego, Medellín 050012, Colombia; abustamante.eye@gmail.com; 4 Grupo Biología y Control de Enfermedades Infecciosas, Universidad de Antioquia, Medellín 050010, Colombia; geysson.fernandez@udea.edu.co

**Keywords:** keratoconus, omics, inflammation, transcriptomic, exposome, genetics

## Abstract

Keratoconus (KC) is a progressive ocular disorder marked by structural and functional alterations of the cornea, leading to significant visual impairment. Recent studies indicate that these corneal changes are linked to molecular and cellular mechanisms that disrupt and degrade the extracellular matrix. This degradation is influenced by proteinases that contribute to a loss of homeostasis and an imbalance in the antioxidant/oxidative state within the cornea, fostering oxidative stress, inflammation, and apoptosis. Although these biological processes have been identified primarily through molecular biology research, omics technologies have significantly advanced our understanding of the physiological and pathological phenomena associated with KC. Omics studies encompassing genomics, transcriptomics, proteomics, epigenomics, and metabolomics, have emerged as critical tools in elucidating the complex biological landscape of various diseases, including ocular conditions. The integrative application of these studies has demonstrated their potential in personalizing medicine across diverse fields such as oncology, neurology, and ophthalmology. This review aims to describe findings from omics research applied to keratoconus, highlighting the genomic, transcriptomic, proteomic, epigenomic, and metabolomic aspects derived from ocular and other biological samples. Notably, the molecular insights gained from these studies hold promise for identifying biomarkers of keratoconus, which could enhance diagnostic accuracy and therapeutic strategies. The exploration of these biomarkers may facilitate improved management and treatment options for patients, contributing to personalized care in keratoconus management.

## 1. Overview of Keratoconus

Keratoconus (KC) is a progressive, degenerative disorder of the cornea characterized by its thinning and conical protrusion [1]. Its clinical manifestation includes changes in corneal curvature, irregular astigmatism, and symptoms such as visual impairment and increased sensitivity to light [2].

The global prevalence of KC ranges from 0.1 to 2% [3,4]. The variability in reported prevalence rates can be attributed to differences in study design, diagnostic criteria, and the populations examined [5]. In addition, the association of KC with specific demographic factors, such as age, gender, and family history, has been observed [6]. Recent studies, such as that by Marx-Gross et al., have revealed a prevalence of 0.49% s in Gutenberg, an increase ten times higher than previously reported in the literature [7]. In Colombia, Mejia-Salgado et al. reported an incidence rate of 10.36 per 100,000 habitants which peaks in males in their early 20s and in females in their late 20s [8].

KC is influenced by a complex interplay of genetic, environmental, and biochemical factors [9]. While genetic predisposition plays a crucial role, numerous studies highlight the multifactorial nature of KC, where both endogenous and exogenous factors contribute to its development and progression [10,11,12]. This multifactorial influence underscores the significance of the exposome, encompassing all environmental exposures, on KC pathogenesis [13,14].

Advances in molecular biology have significantly expanded our understanding of the molecular basis of human diseases, including KC. The introduction of next-generation sequencing (NGS) and multi-omics approaches, such as genomics, transcriptomics, proteomics, epigenomics, and metabolomics, has enabled researchers to delve deeper into the complex biological processes and pathways involved in disease pathogenesis [15,16]. These cutting-edge technologies have provided unprecedented insights into the molecular-level of KC pathophysiology, revealing the intricate interplay of genetic, transcriptomic, proteomic, and metabolomic factors that contribute to the development and progression of the disease [17].

The integration of multi-omics data has enabled the discovery of novel biomarkers and potential therapeutic targets in KC [18]. By examining differential gene expression, protein profiles, and metabolite levels between healthy and pathological corneas, researchers have identified crucial molecular signatures associated with the condition [19,20]. Furthermore, understanding the underlying molecular mechanisms has opened avenues for drug discovery, allowing researchers to develop therapies that specifically target dysregulated pathways and pathological processes in KC.

Therefore, this review aims to explore the application of integrated multi-omics data analysis in KC research. We begin by providing an overview of the biological mechanisms involved in KC. We then summarize the multi-omics approaches utilized in studying the pathogenesis, diagnosis, and analysis of samples of KC. Finally, we discuss the current challenges and future directions of multi-omics research in KC, highlighting its potential to transform clinical practice.

A systematic literature search was performed using databases such as PubMed, Scopus, and Google Scholar. Keywords related to genetics, omics, and keratoconus were used to identify relevant studies. Initial screening was conducted based on titles and abstracts to assess the relevance to the research topic. Full-text articles were subsequently reviewed to ensure they met the inclusion criteria. The inclusion criteria were as follows: (1) studies published in peer-reviewed journals, (2) studies published in English, (3) studies focusing on omics and their relationship with keratoconus, and (4) studies that captured the most recent developments in the field. The exclusion criteria were (1) studies not related to ocular diseases and (2) studies with incomplete data or unclear methodology.

## 2. Physiopathology of Keratoconus

### 2.1. Genetic and Epigenetic Factors

To comprehend the factors that may initiate changes associated with KC and its progression, numerous studies have been conducted through genetic and genomic analyses [21,22]. These analyses have revealed the impact that certain genes have on KC development, its progression and prognostics [22,23,24]. For instance, Bisceglia L et al. identified through genotyping that the chromosomal region 5q21.2 could be a locus of interest in KC [25].

Genome-Wide Association Studies (GWAS) have identified several Single Nucleotide Polymorphisms (SNPs) in candidate genes associated with KC, with notable examples including *HGF*, *NUB1*, *IL-1B*, *CHD11*, *COL27A1*, and *IL1B* [22,26]. The *IL-1* superfamily genes are positioned in a tandem cluster on chromosome 2q14. Their location in regulatory regions suggests they may influence IL-1 protein production by directly affecting transcription, potentially stimulating keratocyte apoptosis in the cornea [27]. This genetic approach provides valuable insights into the functional roles of specific genes in KC, and it holds considerable promise for the development of predictive tests for familial forms of the disease, potentially enabling early intervention and personalized treatment strategies.

Several familial KC reports suggest the importance of genetics in the pediatric population with KC, particularly considering its impact on ocular tissue growth and its genetic role in corneal changes [28]. A study by Tyynismaa H et al. found through genetic mapping in 20 Finnish families that the chromosomal region associated with KC is 16q22.3-q23.1 [29]. Similarly, in the Italian population, Brancati F et al. found the autosomal dominant KC present in members of a family, with its locus attributed to the chromosomal region 3p14-q13 [30]. On the other hand, Nejabat M et al. investigated mutations of visual system homeobox 1 (*VSX1*) and superoxide dismutase 1 (*SOD1*) in KC patients in the south of Iran. The researchers found mutations in different exons in *VSX1* and intron of *SOD1* [31]. Curiously, *VSX1* influenced the keratocyte’s corneal differentiation; it is considered important in ocular development and is particularly involved in the developing cornea. As a result, it plays a significant role in wound healing [25,32]. In addition, Zhang J et al. demonstrated that the variant p.G342E of *VSX1* is implicated in the pathogenesis of KC with an autosomal dominant inheritance pattern and variable expression in the clinical phenotype [33,34].

Other genes implicated in the familial development and progression of KC include single-nucleotide polymorphisms (SNPs) found in intronic and exonic regions of *LOX*, *FOXO1*, *DOCK9*, and *GALNT14* genes [14]. As a result, molecular advancements and genomic analyses of ocular samples have been conducted to identify genetic components and provide a more precise identification of candidate genes associated with KC. These efforts aim to facilitate the development of gene-based therapies, as seen in other ocular pathologies [35].

Currently, the impact of the environment on genes is relevant in the comprehension of health and diseases [36]. This gene–environment interaction can be crucial in understanding the KC disease’s pathophysiology [14]. For example, some studies indicate that environmental factors like air pollution and diet could induce oxidative changes in corneal tissue, potentially leading to alterations in the extracellular matrix, inflammation, and apoptosis [37,38,39]. This explains why KC development is not always bilateral and symmetrical. Indeed, some studies highlight that the clinical presentation of KC can occur in one eye only, influenced by non-genetic factors.

Part of the gene–environment interaction can be explained through epigenetics, which refers to modifications in gene expression that occur without altering the underlying genomic sequence [40]. This modulation can result from various factors, both internal and external, such as pollutants, stress, medications, diet, and lifestyle choices, all of which influence gene expression. It is well established that dietary factors can lead to DNA methylation, potentially silencing protective genes and activating oncogenes. Ocular diseases, including age-related macular degeneration (AMD), glaucoma, and other pathologies, have been studied in the context of epigenetics and its interaction with environmental factors.

Several elements are involved in epigenetics, including histone modifications, DNA methylation, and the activity of small non-coding RNAs (microRNAs, RNAlong, short, RNAi) [40]. Research focusing on microRNA (miRNA) analysis demonstrates some candidates in KC, including miR-184 linked in corneal epithelial cell proliferation and miR-204 that influence corneal wound healing and miR-184 [41,42]. In addition, Kather JN et al. identified miR204 in a murine model as a regulator of proangiogenic factors like angiopoietin-1 [43]. In general, exploring the influence of miR on KC helps in understanding the disease and improving its diagnosis and treatment, which is an aspect studied in therapy for dry eye [44].

DNA methylation is another epigenetic mechanism, and it is associated with several disorders in the eye, including in the posterior segment in diabetic retinopathy and ocular surface disorders such as dry eye and KC [38]. A study published by Kabza M et al. revealed different patterns of hypo- or hypermethylation in *WNT5A*, and this encodes WNT ligands [45]. The WNT signaling pathway is involved in biological processes linked to adhesion, proliferation, and migration cell, which are deregulated in KC [46]. Interestingly, Cuellar G et al. discovered that a WNT10A variant increased the risk of KC associated with reduced corneal thickness [47]. Although many gaps remain in understanding the epigenetic fingerprints contributing to KC pathogenesis, analyzing the corneal epigenome has the potential to revolutionize and even identify possible targets for diagnosis and treatment.

### 2.2. Inflammation, Oxidative Stress, and Cell Death

The development and progression of KC is not clear [48]. Some studies discuss the role of inflammation in this disease [1,13,49]. Moreover, there are biologics mechanisms implicated in KC, such as oxidative stress and apoptosis, which are discussed in this section. For instance, a study by Nichani et al. found elevated levels of metalloproteinase-9 (MMP-9) in the corneas of KC patients, with levels correlating to the severity of corneal ectasia as determined by curvature measurements [50]. Additionally, Loh and Sherwin demonstrated higher levels of inflammatory cytokine proteins in KC corneas [51]. These researchers enhance our understanding of inflammation’s role as a marker of KC disease.

Stromal changes have been reported in KC resulting in alterations in corneal curvature and blurred vision [52,53]. These changes are modulated for proteolytic enzymes like collagenases, metalloproteinases, and general proteinases, which induce reorganization of proteoglycan and extracellular matrix components [37,54]. Shetty R et al. analyzed the cytokine secretion by corneal epithelial cells in KC. They found an upregulation of MMP9, TNFα, and IL6 in both cornea samples and tears from KC patients. Interestingly, the level of these molecules reduced significantly after cyclosporine A treatment [55]. Hence, the role of inflammation in KC has garnered significant attention.

Consequently, polymorphisms, such as those in the *IL-1B* gene, have been linked to an increased risk of keratoconus [56]. Variants in this gene can influence the production of interleukin-1 beta, a key pro-inflammatory cytokine that may contribute to the inflammatory processes and up-expression of MMP9 observed in keratoconus [57].

In various diseases such as KC, oxidative stress and inflammation are intricately intertwined with pathophysiological processes [58]. Specifically, in ocular conditions, one process often triggers or exacerbates another [59]. The mechanism by which inflammation leads to corneal thinning, especially at the center, is unclear [50]. Studies in inflammation models suggest that external factors can activate dendritic cells, leading to the release of T lymphocytes that secrete inflammatory mediators like cytokines, chemokines, and proteases, which degrade the extracellular matrix [37,60,61]. This process also leads to the release of reactive oxygen species, ultimately deregulating the antioxidant system and activating the oxidative stress cascade [59]. Nevertheless, certain studies propose an alternative scenario, suggesting that oxidative stress may initiate and trigger the release of reactive oxygen and nitrogen species (ROS/RNS) [60,62]. These, in turn, activate transcription factors, including NF-κB, thereby influencing the activation of inflammatory genes [63]. Thus, an important biological feature of KC is the participation of oxidative stress and inflammation.

**Figure 1:**
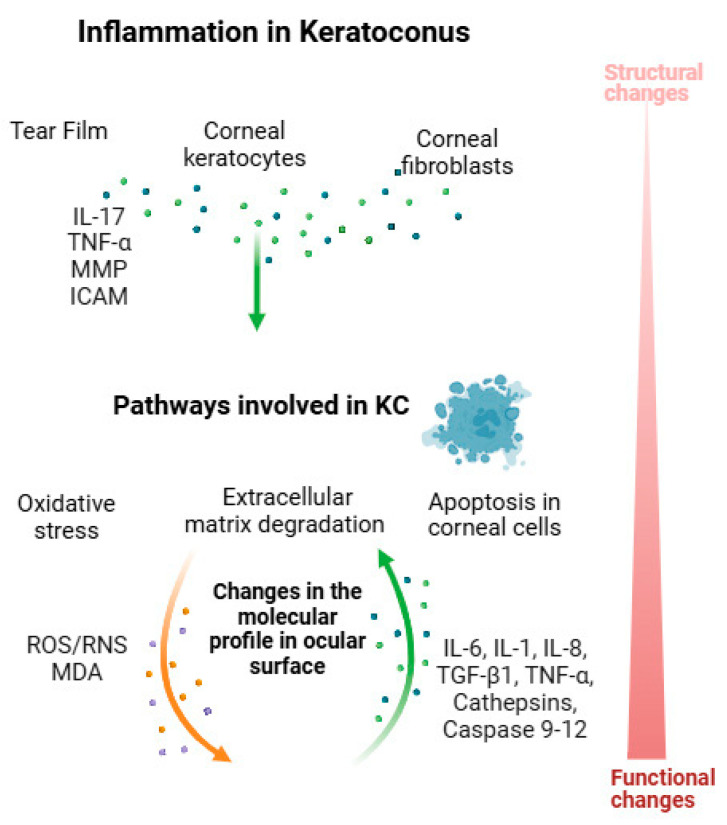
The schematic summarizes the role of inflammation in keratoconus (KC), highlighting changes in molecular components of the tear film and corneal cells (keratocytes and fibroblasts). These alterations contribute to the dysregulation of molecular pathways, including oxidative stress, extracellular matrix degradation, and apoptosis.

In KC, an important focus lies on the morphological and functional changes in cells such as epithelial cells, fibroblasts, and keratocytes [64]. Both inflammation and oxidative stress within these cells modulate signaling pathways that ultimately lead to cell death processes like autophagy and apoptosis [65,66]. A study isolating stromal cells from KC corneas revealed that the overexpression of TIMP-1 induced morphological changes, and subsequently led to cellular apoptosis [67]. Similar findings were recently reported in blood serum by Nowak-was et al. [68]. This finding is supported by Kaldawy et al. who, using the TUNEL assay, demonstrated an increased number of cells with DNA fragmentation in KC corneas compared to non-KC corneas [69]. In conclusion, clinical findings may represent molecular changes mainly associated with oxidative stress, inflammation, and corneal cell death (Figure 1).

## 3. Multi-Omics in Keratoconus

### 3.1. Importance of Omics Technologies in Medicine

Omics refers to the comprehensive study of biological molecules on a large scale, providing insights into health and disease at a molecular level [15]. This interdisciplinary approach includes genomics, proteomics, metabolomics, transcriptomics, and other -omics disciplines, each focusing on different aspects of biological molecules such as genes (genomics), proteins (proteomics), metabolites (metabolomics), and RNA transcripts (transcriptomics) [70]. Omics technologies have significantly advanced the exploration of complex interactions within biological systems, offering a comprehensive view of cellular processes and their dysregulation in diseases [71]. Currently, biomedical research is focused on comprehending biological events in both health and disease.

Research in omics has revolutionized our understanding of disease mechanisms, biomarker discovery, personalized medicine, and therapeutic targets [71]. The concept of personalized medicine refers to an approach to medical care and research that considers individual differences in genetics, environments, and lifestyles [72]. It aims to tailor medical decisions, diagnostics, interventions, and therapies to the individual patient based on their unique characteristics [73]. Similarly, omics research includes advantages in clinical practice, particularly in the early detection of diseases [73]. By analyzing large datasets generated from omics studies, researchers can identify molecular signatures associated with diseases, predict patient outcomes, and tailor treatments based on individual genetic and molecular profiles [15]. Therefore, the integration of omics data with clinical and epidemiological information has enhanced our ability to unravel the complexities of human health and disease, paving the way for innovative diagnostic tools and precision medicine approaches.

Moreover, the advent of NGS offers significant advantages, enabling the rapid analysis of a wide spectrum of genetic, transcriptomic, and other omics data such as proteomics and lipidomics [74]. This facilitates the identification of biomarkers for pathology, thereby aiding in the discovery of therapeutic targets [75]. While omics technologies complement traditional molecular biology analyses, such as gene panel studies, transcript expression profiling, and protein analysis using techniques like Western blot and immunofluorescence, they do not seek to replace them entirely.

In brief, omics studies represent a significant advancement in our comprehension of disease, with personalized medicine emerging as a pivotal approach. This approach acknowledges the invaluable insights gain from omics, facilitating tailored diagnoses and treatments, particularly evident in conditions like cancer [76], infectious diseases [77] and ocular diseases such as glaucoma [78], age-related macular degeneration [79], and ocular surface disorders such as dry eye [80] and KC [18].

### 3.2. Omics Approaches to Understanding Keratoconus at the Molecular Level

Although some genes and pathways are characterized as responsible for KC, many unknown molecular mechanisms are still involved in this condition. Currently, the availability of omics technologies to acquire genome-wide data is expected to change the way we formulate and address biological questions. With nearly all genes in hand, the conventional reductionist approach in the study of KC—studying one gene at a time—can now be complemented by more global or integrative approaches that consider all genes at once [74]. Even though reductionist approaches have revealed many fundamental aspects of biology, they are limited in providing a comprehensive picture of the life of cells, tissues, and organisms. Thus, it is reasonable to imagine that more integrative genome-wide approaches will bring a better understanding of the KC processes at a fundamental level. So, by integrating the information contained in the genome-wide datasets, increasingly meaningful biological hypotheses can be formulated. However, it should be kept in mind that these hypotheses still need to be tested in the context of relevant biological settings, perhaps using more refined approaches.

### 3.3. Genomics

Genomics is a part of omics analysis, and this area emphasizes the structure, function, evolution, mapping, and editing of genomes. A genome is the complete set of genetic material (DNA) within an organism [71]. Genomics involves studying the entire DNA sequence of an organism and understanding how genes function and interact to influence traits and behaviors. The genomics analyzed included genetic variation, comparative genomics to identify protected regions, and genome evolution [15]. Therefore, identifying genes associated with diseases provides opportunities for early diagnosis, as evidenced in conditions like cancer and other diseases [74].

Genetic and genomic analysis are related to the biological comprehension of processes from the information of genes [71]. However, genetics refers to the study of a single gene at a time, individual genes, or specific regions of DNA, to understand their inheritance patterns, mutations, and effects on traits. This type of analysis includes variations within genes (such as mutations or polymorphisms) and their association with specific phenotypes, and is commonly used in studies of Mendelian inheritance patterns [81]. In contrast, genomic analysis involves studying the entire genome of an organism, which includes all the genetic material (DNA) [82]. This analysis includes the study of single nucleotide polymorphism (SNP), which may have no negative effect on the phenotype (e.g., nonsense variant). Exome analysis can result in insights into various diseases, and it has enhanced our knowledge of how human genes interact with each other and the environment to influence health [83].

The genes involved in KC include those associated with cellular proliferation, immune response, and injuries in extracellular matrix [1]. For instance, in whole exome sequencing, researchers identified polymorphism in *ADAMTS16* (c.1314-5>TC), *COL23A1* (c.960+3>A), *CD248* (c.1323_1325del), and *WNT16* (c.1_2insCCCA) [18] (Table 1). Interestingly, these genes are related to biological processes such as cell proliferation and migration, WNT signaling, collagen catabolic process, and extracellular matrix remodeling [45,47]. Thus, understanding the multiple genes involved and their biological processes provides a foundation for grasping the complexity of KC disease.

In GWAS analysis, Table 1 reveals multiple gene regulators of transcription factors associated with the extracellular matrix. For example, FOX1 (rs2721051) is implicated in the maintenance of keratinocyte stem cell identity. Evidence shows that SNPs associated with KC often alter the methylation of many genes, including *LOX*, *PDDC1*, *SMAD3*, *HOXB1*, *KLF5*, and *BANP* [84,85]. Therefore, genomics analysis has revealed DNA variants related to Mendelian and non-Mendelian inheritance patterns that could be associated with corneal modifications.

Peripheral blood mononuclear cells (PBMCs) are commonly analyzed in genomic studies [86]. While the use of peripheral cells is widespread, exploring corneal and conjunctival cells through impression cytology and tear film analysis could serve as a valuable tool in identifying new genomic targets.

Among the most relevant genomic biomarkers are those associated with alterations in the genes *IL1B*, *CDH11*, *FOXO1*, *COL5A1*, *FNDC3B*, *ZNF469*, *LOX*, *PNPLA2 COL27A1*, *COL6A2*, *TGFBI*, and *VSX1*.

Several years ago, techniques were limited to analyzing only a small number of genes at a time. However, with the introduction of genomics into ophthalmology, it is now possible to develop a genetic profile that not only aids in the diagnosis of KC, but also allows for monitoring and correlating genetic results with clinical findings.

**Table 1:** List of genomics studies of keratoconus.

Type of Analysis	Methodology Employed	Population Age (Mean)	Sample	Findings	Reference
Whole Exome Sequencing	PBMCs	55.5 ± 0.5	KC	Eight candidate genes (*COL23A1*, *CD248*, *ADAMTS16*, *ADAMTS3*, *COL4A3*, *COL18A1*, *WNT16*, and *COL6A2*).	[18]
GWAS	PBMCs	57 ± 8	Control/KC	The authors identified several CRF and CCT loci harbored within 1 Mb of mendelian genes associated with rare corneal or connective tissue diseases. These included *FECD7*, *TCF4*, *SLC4A11*, *UBIAD1*, *MPV17*, *ZNF513*, *COL5A1*, *ZNF469*, *COL8A2*, *AGBL1*, *SMAD3*, *DCN*, *KERA*, and *TGFB2*.	[87]
GWAS	PBMCs		Control/KC	Six loci were previously associated with keratoconus (*FOXO1*, *COL5A1*, *FNDC3B*, *ZNF469*, *LOX*, and near *PNPLA2*), and these were associated with central corneal thickness. In addition, strong associations were found near or within genes that code for fibrillar collagens (types I and V), microfibrillar (VI), and peri-fibrillar (XII) structures, implicating impaired cohesion of the collagen matrix in the pathogenesis of keratoconus.	[84]
GWAS	PBMCs	56.4 ± 13.0	Control	STON2 rs2371597 showed a significant association with corneal central thickness. On the other hand, STON2 rs2371597 C allele showed increased CCTs in a Latino population.	[86]
GWAS	GitHub repository (last updated in May 2019)		GWAS for central corneal thickness (CCT) and corneal resistance factor (CRF)		[26]

All findings presented in this table are based on studies of genomic associations with keratoconus. GWAS: Genome-Wide Association Study. PBMCs: Peripheral Blood Mononuclear Cells. KC: Keratoconus.

### 3.4. Transcriptomics

The transcriptome represents the complete repertoire of RNA transcripts within an organism, tissue, or cell at a particular point in time or under specific conditions. This complex collection comprises messenger RNAs (mRNAs), which encode proteins, as well as non-coding RNAs and small RNAs, which play crucial roles in regulating gene expression and other cellular processes [15].

Transcriptomic studies involve analyzing and quantifying RNA molecules to understand gene expression patterns, regulatory mechanisms, and functional pathways within cells or organisms [88].

Transcriptomics studies have provided insight into biological events associated with both normal physiological conditions and, more prominently, pathophysiological events [15]. Gene expression under specific conditions or moments plays a crucial role, acting as a signal for changes in cellular phenotype, cell signaling, and organelle modification [89]. This modulation of gene activity, where genes can be activated or suppressed and their expression levels adjusted, constitutes the essence of transcriptomics.

Common types of transcriptomic analysis include RNA sequencing (RNA-seq), microarrays analysis, single-cell RNA sequencing (scRNA-seq), and RNA-seq for non-coding RNAs that include microRNAs (miRNAs), long non-coding RNAs (lncRNAs), and small nucleolar RNAs (snoRNAs), are used to study their roles in gene regulation and cellular processes [90]. Due to the significant impact of gene expression levels on biological processes associated with health and disease, technologies such as microarrays and RNA-seq have become essential tools [91]. Microarrays involve hybridizing DNA sequences onto a chip, where the emitted fluorescent signal quantifies gene expression [92]. In contrast, RNA sequencing (RNA-seq) enables precise measurement of gene expression levels and identification of alternative splicing events, providing substantial advantages over microarray technology [90]. This analysis has enhanced our understanding of the KC transcriptome (Table 2).

As previously mentioned regarding the role of inflammation in the progression of KC [95], transcriptomic analysis has demonstrated the impact of immune response in early stages of diseases [91,96]. Sun X et al. described the association between immune and inflammatory genes and pathways with KC. They found 547 genes involved in some biological processes, including leukocyte cell–cell adhesion, cytokine receptor activity, endopeptidase activity, and cellular and humoral immune response [16]. Across various studies, there is consistency with Shinde V et al., who revealed from corneal samples that downregulated genes were in different pathways. These included pathways related to collagen synthesis and modification, IL6 signaling, as well as to extracellular matrix organization and downregulated genes involved in Transforming Growth Factor-Beta (TGF-β) signaling pathways (a regulator of inflammation) [92].

The pathogenesis of KC involves oxidative stress and inflammation [64,97]. Although the definition of inflammation is debated, transcriptomic analysis has confirmed the differential expression of genes related to oxidative stress, extracellular matrix remodeling, the MAPK, and TGF-β pathway, which play a crucial role in inflammatory responses [94,98]. Furthermore, tear film and corneal sample analysis in KC patients have validated the inflammatory role of RNA sequencing. Single-cell RNA sequencing (scRNA-seq) analysis has underscored the impact of cytokines and matrix metalloproteinases 1–14 [99]. Indeed, integrated transcriptomic and bioinformatic analyses have identified predictive biomarkers associated with the inflammatory profile at different stages of KC related to protease production in corneal stromal cells [96]. These biomarkers are linked to ocular surface immune cells and the inflammatory profile, suggesting potential therapeutic targets for managing KC [100]. Subsequently, the clinical approach should incorporate a more comprehensive assessment of the ocular surface to identify early immune dysregulation that correlates with the severity of KC, as confirmed by corneal topographic indices.

Based on RNA sequencing analyses, a transcript profile could serve as a potential future biomarker, with gene dysregulation observed in COL1A1, COL1A2, MMP-1, MMP-9, MMP-2, TGF-β1, IL-6, TNF-α, LAMA1, LOX, TIMP-1, and SOD1. These genes are involved in pathways related to collagen synthesis, matrix metalloproteinases, transforming growth factor-beta (TGF-β), pro-inflammatory cytokines, extracellular matrix (ECM) remodeling, and tissue homeostasis.

In conclusion, transcriptomic studies have provided new insights into the biological mechanisms underscoring KC pathogenesis. Furthermore, samples analyzed in this omics field, such as conjunctival and corneal tissues, as well as tear film, are proposed as valuable tools in the search for biomarkers of ocular diseases. Therefore, it is crucial to standardize these techniques for use in ocular samples. To illustrate this, Wolf J et al. have developed a platform that integrates transcriptomic databases from various ocular pathologies compared to controls, thereby enhancing our understanding of genes that are either up- or down-regulated across different ocular tissues and samples [101]. This resource enhances essential information for developing diagnostic strategies and treatments based on omics findings.

### 3.5. Proteomics

Proteomic analysis refers to the comprehensive study of proteins expressed by a biological sample at a given time or under specific conditions. This field aims to identify, quantify, and characterize proteins to understand their functions, interactions, modifications, and roles in biological processes [73]. Proteomic analysis involves several techniques such as mass spectrometry (MS), two-dimensional gel electrophoresis (2D-PAGE), protein microarrays, and post-translational modification that includes phosphorylation, glycosylation, acetylation, and ubiquitination [102].

Proteomic analysis has become increasingly valuable due to its ability to elucidate the functional impact of proteins on cellular biological processes [102]. The synergy between transcriptomics and proteomics has led to significant advancements in understanding disease biology. Currently, comprehensive platforms for the human proteome and proteomic data from disease models, such as cancer, are publicly available, offering valuable research resources (https://www.proteomicsdb.org/, https://www.ebi.ac.uk/pride/, accessed on 15 June 2024).

Proteomic analysis plays a crucial role in advancing our understanding of cellular mechanisms, disease processes, drug discovery, and personalized medicine by providing insights into the complex protein networks that govern biological systems [66]. By identifying differentially expressed proteins and their interactions, proteomic studies can elucidate the molecular pathways underlying disease pathogenesis [102]. This knowledge can guide the development of targeted therapies and the identification of novel biomarkers for the early diagnosis and monitoring of disease progression.

In recent years, the rapid progress in the field of vision science has underscored the significance of tear fluid not only in analyzing proteins linked to ocular disorders but also in revealing insight into various systemic diseases such as diabetes [103], neurodegenerative disorders [104], and cancer [105]. Therefore, the importance of the tear proteome is emphasized, both technically for its non-invasive nature compared to other bodily fluids and due to its proximity to the nervous system, offering insights at both proximal and distal levels [106,107,108,109].

In the cases of KC, protein analysis studies have elucidated the role of inflammation and other events in its pathogenesis [110]. However, proteomics offers several advantages over traditional techniques such as ELISA (Enzyme-Linked Immunosorbent Assay) and Western blotting for protein analysis [111]. For instance, proteomics allows for the simultaneous analysis of thousands of proteins within a sample, providing a comprehensive view of the proteome. Additionally, proteomics techniques, such as mass spectrometry-based approaches, offer precise quantitative analysis of protein abundances over a wide dynamic range. Finally, modern proteomics methods are highly sensitive, capable of detecting proteins at low concentrations in complex biological samples, an important aspect in ocular samples like the tear film [108,109] (Table 3).

The lacrimal proteome in KC indicates the involvement of oxidative stress, inflammation, and some biological processes in its development [112,114]. Interestingly, a comparison of ocular samples from patients with allergic conjunctivitis (AC) and KC has revealed a shared inflammatory profile and alterations in protein expression, confirming the association between allergic process and KC [114]. This demonstrates the close relationship between the conjunctival allergic process, its inflammatory triggers, and the inflammatory profile, which can lead to the loss of the corneal extracellular matrix and the development of KC.

As a result, these findings underscore the importance of considering the inflammatory landscape in the clinical management of KC [112,116]. Indeed, the advantages of proteome analysis in KC highlight the role of inflammatory events as indicators of disease severity, consistent with transcriptomic findings [115]. This aligns with the impact of KC management strategies, such as contact lens wear and surgical interventions, on disease progression.

Other biological processes have been discovered in these proteomic studies. Lopez-Lopez et al. identified proteins as hemopexin, annexins, vitamin D binding protein, and tubulin α1C chain, and biological pathways involving differential proteins like actin cytoskeleton organization, interleukin-12 signaling pathway, apoptotic process regulation of wound healing, and regulation of vesicle fusion [112]. Furthermore, Lema I et al. revealed a differential pattern in proteins: under-expressed proteins included IGKC, ZAG, and lactoferrin, though their role in KC is unclear [113]. In contrast, Shinde V et al. suggest the role mTOR signaling [115], which is crucial for regulating cell growth, proliferation, and apoptosis in response to nutrient availability [117]. Consequently, studies indicate that keratoconus-derived cells have altered ECM deposition and composition, which may be influenced by mTOR signaling [62,118]. Therefore, targeting the mTOR pathway may provide new therapeutic approaches for this disease.

According to proteomic analyses of ocular samples from individuals with KC, it is possible to propose a proteomic profile involved in the pathogenesis of this disease, which includes collagen types I and IV, fibronectin (FN1), vitronectin, MMP-9, TIMP-1, IL-6, TNF-α, annexin, SOD1, GPX1, KRT3, decorin, and HSP70.

In brief, proteomics research in KC has identified potential biomarkers, elucidated disease mechanisms, highlighted therapeutic targets, and revealed the peripheral involvement of non-cone regions in disease pathogenesis. Continued efforts to translate these findings into clinical practice could significantly improve the management of KC.

### 3.6. Epigenomics

Epigenetic analysis is the study of epigenetic modifications and their effects on gene expression and cellular function. It involves the assessment of various epigenetic mechanisms such as DNA methylation, histone modifications, chromatin structure, and non-coding RNA regulation [119]. Epigenetics plays a crucial role in human health and disease [120]. Epigenetic mechanisms, such as DNA methylation, histone modification, and microRNA regulation, can produce heritable changes in gene expression without altering the DNA sequence [121]. These changes have been incorporated into the physiopathology of diseases like cancer [122], viral infection [123], and ocular disorders [124].

Epigenetics plays a crucial role in various ocular disorders, including KC [38]. Alterations in DNA methylation, histone modifications, and non-coding RNAs can modulate the expression of genes involved in inflammation, oxidative stress, and extracellular matrix remodeling [125,126]. These epigenetic mechanisms have been implicated in the pathogenesis of KC [127,128]. For example, studies have profiled miRNA expression in human ocular tissues, such as the cornea, ciliary body, and trabecular meshwork, using miRNA sequencing [129,130]. The dysregulation of specific miRNA profiles has been associated with conditions such as glaucoma [129] and KC [131].

In particular, Kalaimani et al. identified 62 miRNAs differentially expressed in corneal epithelial stem cells, including miR-21-5p, miR-3168, miR-10a-5p, miR-150-5p, miR-1910-5p, and miR-143-3p [132]. These miRNAs have been shown to regulate inflammatory responses, proliferation, cell differentiation, and AKT signaling—processes that are crucial in the development and progression of KC [133]. Overall, the application of epigenomic approaches has the potential to uncover key epigenetic regulators involved in the pathogenesis of KC (Table 4). The identification of these epigenetic modifications could lead to the development of new diagnostic biomarkers and targeted therapeutic interventions.

The WNT signaling pathway plays a crucial role in various cellular processes, including cell proliferation, differentiation, and tissue development [45]. In KC, dysregulation or aberrant activation of WNT signaling may contribute to the structural changes and biomechanical abnormalities observed in the cornea [134]. Indeed, genomics and transcriptomic analysis revealed one of the main gene targets is *WNT* [46,86]. Additionally, in epigenomic analysis, Kabza M et al. identified 112 methylated regions (DMRs) with distinct patterns of DNA hypermethylation (43 regions) or hypomethylation (69 regions) located in regulatory regions associated with CpG islands [45]. Notably, they observed that two DMRs located on chr3:55,474,327-55,474,562 and chr17:46,769,843-46,769,996 overlapped the exons of WNT family member genes, *WNT5A* and *WNT3*, respectively.

miRNAs are considered crucial regulators of gene expression at the post-transcriptional level and have been implicated in numerous physiological and pathological processes [135]. Their dysregulation has been associated with various diseases, including cancer [136], cardiovascular disorders [137], neurodegenerative diseases [138], metabolic disorders [139], and ocular surface disorders [140]. Stachon T et al. identified dysregulation of miR-180 in epithelial cells and miR-379 in stromal cells [94]. The most upregulated miRNAs in the epithelium or stroma corneal were miR-211-3p and miR-135b-5p, respectively, likely associated with corneal neovascularization, and the downregulated miRNAs were miR-634 and miR-936 in the epithelium and stromal KC (miR-936). In contrast, Zhang Y et al. identified differentially expressed miRNAs in the aqueous humor of KC patients: miR-222-3p, miR-363-3p, and miR-423-5p. These miRNAs influence antigen processing and presentation, endocytosis, mismatch repair, and Hippo signaling [141]. These results underscore the importance of exploring miRNAs as biomarkers and regulatory elements in understanding KC pathogenesis.

In addition, Drewry M et al. profiled miRNA expression by miRNA-seq in human ocular samples (cornea, ciliary body, and trabecular meshwork) and alterations in this profile could indicate conditions like glaucoma and KC [129]. Kalaimani et al. identified 62 miRNAs, including miR-21-5p, miR-3168, miR-10a-5p, miR-150-5p, miR-1910-5p, and miR-143-3p, which notably modulate inflammation response, proliferation, cellular differentiation, and AKT signaling in corneal epithelial stem cells (CESCs) [132].

Considering the exploration of microRNAs as potential biomarkers, miR-184, miR-211-3p, and miR-936 could be studied in depth as epigenetic biomarkers as they are involved in corneal epithelial differentiation, wound healing, extracellular matrix (ECM) remodeling, collagen synthesis, fibrosis, and ECM organization. Since their association with KC is relatively recent, it would be interesting, with the advancement of newer technologies, to determine at which stage of the disease they could be useful as biomarkers.

The epigenomic analysis of ocular samples, including corneal tissues, tears, and aqueous humor, is crucial for identifying potential biomarker panels. The techniques used in these low-volume samples have led to significant advancements in the field. Recently, microRNAs (miRNAs) have emerged as promising therapeutic agents for various ocular disorders, underscoring their potential in future diagnostic and treatment strategies [142]. Ongoing research and database analysis of miRNAs may further deepen our understanding and application of these biomarkers in clinical settings.

### 3.7. Metabolomics

Metabolomics studies refer to the systematic analysis of small molecules, known as metabolites, within biological samples such as cells, tissues, or biofluids. Metabolomics aims to comprehensively identify, quantify, and study the metabolic products and pathways involved in cellular processes and biochemical reactions [143]. Techniques such as mass spectrometry (MS) and nuclear magnetic resonance spectroscopy (NMR) are used to quantify metabolites, providing information on their concentration levels and changes in response to different conditions [144].

All metabolites studies are intricately connected to biological processes and metabolomics analysis offers critical insights into how environmental factors interact with health and disease, illuminating exposure–response relationships [143]. Currently, epidemiological research is using metabolomic studies to comprehend the interaction between environmental exposure and animal health, human health, and the ecosystem [145]. As discussed below, the exposome holds relevance in clinical practice, particularly in ocular disorders like KC [146].

Metabolomic studies contribute to advancing personalized medicine, drug discovery, nutritional science, and understanding environmental impacts on health by providing a holistic view of metabolic processes and their implications in health and disease [77]. Interestingly, metabolomic analysis has identified upregulated metabolites associated with energy production, amino acid metabolism, and lipid metabolism both in ocular samples (corneal and tear) and in serum (Table 5). These findings have indicated alterations in metabolic pathways, such as the tricarboxylic acid (TCA) cycle, which is crucial for cellular energy—a factor essential for corneal transparency and cellular activity [60].

As a consequence, in corneal diseases, metabolomic profiling through techniques such as nuclear magnetic resonance (NMR), mass spectrometry (MS), liquid chromatography-quadrupole-time-of-flight mass spectrometry (LC-Q-ToF/MS), and gas chromatography and mass spectrometry (GC/MS) have showcased changes in physiological events in the cornea such as glycolysis and glutaminolysis via the TCA and Kreb’s cycle under aerobic conditions, and the sorbitol pathway in anaerobic conditions. In the pathogenesis of KC, oxidative stress plays a role in progression and inflammatory activation [147,148].

In conclusion, according to the metabolomic analysis (Table 5), various metabolites are upregulated in KC, including fatty acids, sterols (cholesterol derivatives) and their esters, carboxylic acids (e.g., citric, succinic, and oxalic acids), as well as sugars and their derivatives (glucose, gluconic acid, sorbitol, and myo-inositol). These suggested findings underscore the importance of integrating metabolomic approaches into KC research, as they can elucidate the complex interplay between metabolic dysregulation and the disease’s clinical manifestations and suggest potential metabolomic biomarkers and innovative interventions.

## 4. Translational and Clinical Implications

KC is described as a multifactorial disease characterized by the progressive thinning and conical deformation of the cornea, leading to visual impairment [13]. Its etiology is complex, involving both genetic and environmental factors, which lead to various molecular and cellular changes [12].

Omics studies have identified several key genes and proteins that may serve as biomarkers or therapeutic targets for KC (Table 1). Transcriptomics and proteomics have uncovered differentially expressed genes and proteins related to extracellular matrix [84], TGFβ signaling [100], oxidative stress [92], and apoptosis pathways [116] (Table 2 and Table 3). For example, a reduction in the expression of the LOX gene and protein has been observed in blood samples from keratoconus (KC) patients, and alterations in LOX expression in corneal tissue have also been reported [85,149]. These molecules could potentially be used as diagnostic biomarkers or targets for therapeutic intervention to halt disease progression.

In addition, multi-omics studies, which integrate genomics, transcriptomics, and proteomics, provide compelling evidence that factors from both the innate and adaptive immune responses contribute significantly to tissue and molecular changes in the cornea of individuals with KC [18]. Indeed, thanks to these contributions and conclusive findings, KC is now understood as an inflammatory pathology [16]. Krok M et al. evaluated the levels of cytokines and chemokines in tears from individuals with KC before and after corneal cross-linking (CXL), reporting that the expression profile of pro-inflammatory cytokines was higher in moderate to severe cases, with levels correlating with the analyzed corneal topographic changes [92]. Moreover, gene expression and the protein level of galectin 3, a protein biomarker of KC is modified after CXL [150]. Similar findings have reported the downregulation of fibrosis markers such as collagen III and α-smooth muscle actin (α-SMA) [151].

In addition, multi-omics studies, which integrate sequencing in genomics, transcriptomics, and proteomics, provide compelling evidence that factors from both the innate and adaptive immune response contribute significantly to tissue and molecular changes in the cornea of individuals with KC [18]. Indeed, thanks to these contributions and conclusive findings, KC is now understood as an inflammatory pathology [16]. Krok M et al. evaluated the level of cytokines and chemokines in tears from individuals with KC before and after corneal cross-linking (CXL), reporting that the expression profile of pro-inflammatory cytokines was higher in moderate to severe cases, which correlates with the analyzed corneal topographic changes [97]. These findings, combined with those reported in transcriptomics (Table 2), suggest that inflammation not only plays a role in the development but also in the progression of KC and emphasizes its significance in the clinical approach.

On the other hand, the gene–environment interaction plays an important role in the understanding of ocular diseases [127]. For example, Hong M et al. in a recent review, discussed the significant implications of the exposome on ocular diseases, highlighting the dysregulation of the ocular surface microbiome as a risk factor for the development of KC, dry eye, glaucoma, and allergic conjunctivitis [146].

Alterations in the microbiome may be linked to environmental factors, such as air pollution, with particular emphasis on exposure to particulate matter (PM) [39]. The relationship between PM and allergic conjunctivitis has been well-documented, where PM not only disrupts the microbiome but also influences metabolic and inflammatory pathways. Notably, research has highlighted a strong association between allergic conjunctivitis and the onset of keratoconus (KC) [152,153]. As a result, the integration of epigenomic and metabolomic analyses is increasingly recognized as a powerful tool for diagnosing KC.

Exposure to particulate matter (PM), especially fine particulate matter (PM2.5), has become progressively linked to the pathogenesis of KC. Recent studies indicate that PM exposure may exacerbate established KC risk factors, such as eye rubbing and atopy. For instance, air pollution has been shown to induce alterations in DNA methylation patterns. Specifically, in ectatic corneas, the inhibition of METTL3 significantly reduced the expression of MMP1, suggesting a pathogenic role for METTL3 in the development of KC [154].

Microbial metabolites play a significant role in modulating biological activities that influence immune responses, neuronal activity, and overall health [155]. This suggests that dietary choices can impact systemic and ocular diseases, including uveitis, glaucoma, dry eye, and KC. Lasagni Vitar RM et al. have proposed that nutritional factors, such as minerals and vitamins, along with metabolic factors like hormones and metabolites, may influence redox homeostasis, the resistance of collagen fibrils, and the activation of matrix metalloproteinases (MMPs) [9]. Furthermore, research by Akkaya S et al. established a notable correlation between serum vitamin D levels and the progression of KC [156], while additional studies have identified various dietary factors linked to KC [11,65,118]. These findings highlight the critical clinical implications of vitamins in KC and their potential applications in treatment strategies.

**Figure 2:**
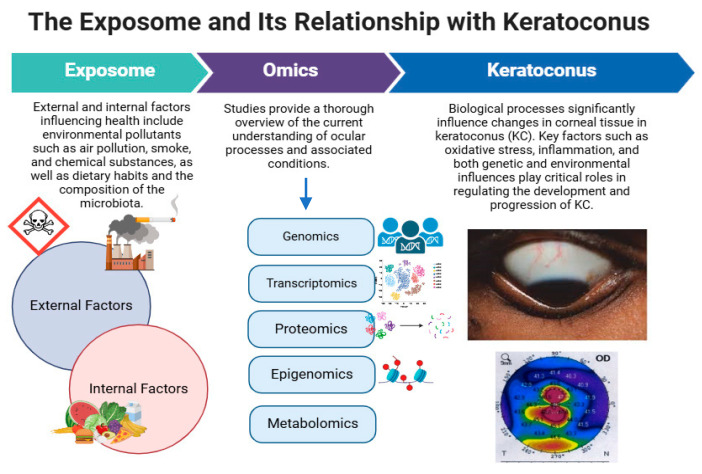
Representation of environmental (internal and external) factors that influence Keratoconus. KC (Keratoconus). The green box represents aspects related to the exposome, while the purple box illustrates the various disciplines of omics. Finally, the blue box describes KC. By integrating these three concepts, a deeper understanding of the disease is achieved.

The interplay between diet and KC underscores the importance of nutritional interventions in managing this complex ocular condition [157]. The evidence suggests that specific vitamins and minerals can influence the disease progression, which opens avenues for dietary modification as a complementary approach to traditional therapies [158]. For instance, optimizing vitamin D levels may not only contribute to overall health but also serve as a protective factor against the worsening of KC [156]. This perspective aligns with the growing recognition of the role of lifestyle factors in the management of ocular diseases, emphasizing the need for a holistic approach that incorporates dietary considerations and their relevance in molecular events associated with KC (Figure 2).

Omics in ophthalmology has emerged as a novel tool for identifying genetic, protein, and metabolic biomarkers specific to various ocular diseases [159]. By analyzing a patient’s omics data, ophthalmologists can gain valuable insights into the molecular basis of conditions such as glaucoma, macular degeneration, diabetic retinopathy, and KC, facilitating personalized medicine and early diagnosis. Additionally, omics technologies have enabled researchers to uncover the molecular and cellular pathways involved in eye diseases, driving the development of new biomarkers and novel therapeutics, particularly in KC, by enhancing the understanding of disease mechanisms.

Incorporating omics technologies into this research can further elucidate the mechanisms by which dietary components affect KC. By employing high-throughput techniques such as metabolomics, epigenomics, and proteomics, researchers can identify specific metabolites, miRNAs and proteins that correlate with dietary intake and disease progression [9,148]. This integrative approach could lead to the discovery of novel biomarkers for early detection and progression monitoring of KC, as well as the development of nutritional and molecular targets [9,160]. Ultimately, understanding the biochemical pathways influenced by dietary and environmental factors could improve clinical strategies for managing KC and enhancing patient outcomes. Therefore, future research on KC should prioritize understanding the role of the *exposome*—an individual’s lifetime accumulation of environmental exposures—in both the development and progression of the disease (Figure 2).

From a technical perspective, while omics technologies such as genomics, proteomics, and metabolomics have shown promise in identifying biomarkers to improve the diagnosis of KC, the immediate implementation of these technologies for routine screening across the entire population, including the pediatric population, presents several challenges. Key factors to consider include the need for standardized protocols, the significant time required for data analysis, and the necessity of specialized equipment and expertise.

Current diagnostic methods for KC primarily rely on clinical examinations and imaging techniques [161,162]. While these approaches are valuable, they often fail to detect early-stage disease or accurately predict its progression [163]. To address these limitations, the integration of omics-derived biomarkers presents a promising opportunity to enhance diagnosis and management strategies for KC, providing more personalized and effective treatments for this complex corneal disorder. In conclusion, the combination of omics-derived biomarkers and exposome factors holds great promise for revolutionizing the diagnosis and management of KC. By supporting the early identification and more precise monitoring of disease progression, these innovative approaches can ultimately improve patient outcomes and inform targeted treatment decisions.

## Figures and Tables

**Table 2:** List of transcriptomics studies of keratoconus.

Type of Analysis	Methodology Employed	Population Age (Mean)	Sample	Findings	Reference
RNA-seq	Corneal tissue/ Blood samples	20.14 ± 1.81/16.00 ± 2.27	Control/KC	Leukocyte cell–cell adhesion, regulation of lymphocyte activation, adaptative immune response, and humoral immune response.	[16]
qPCR	Corneal epitheliums	28.5 ± 5.7/22 ± 4.2/45 ± 7	Control/KC early/KC advanced	In early and advanced KC, they found downregulation in genes related to oxidative stress. In addition, there was an imbalance in apoptosis, proliferation, and differentiation pathways.	[91]
Microarrays/qPCR	Corneal epitheliums	32.6 ± 9.4/26.6 ± 1.6	Control/KC	The biological processes related with KC were leukocyte migration, cell chemotaxis, response to chemokine, and calcium ion homeostasis.	[19]
RNA-seq	Normal primary human corneal fibroblast (N-HCF) and primary corneal fibroblast cells of II-1 (II-1-HCF)	28 ± 0/55.5 ± 0.5	Control/KC	The analysis revealed collagen catabolic processes, collagen trimer, biosynthesis, assembly of collagen fibrils, and the proteinaceous ECM.	[18]
RNA-seq	Corneal epitheliums	57.4 ± 14.7/30 ± 14.2	Control/KC	IL-6 signaling and immune and inflammatory responses were significant in both patient groups. In addition, NRF2 (regulated oxidative stress response) was significantly decreased in samples.	[92]
RNA-seq	Corneal epitheliums	61.87 ± 12.78/31.87 ± 8.55	Control/KC early/KC advanced	Downregulated genes were in different pathways: including pathways related to collagen synthesis and modification, as well as extracellular matrix organization and focal adhesion. In addition, they found downregulation in TGF-β and WNT signaling pathways.	[46]
Microarrays/qPCR	Human corneal epithelial (HCE) and human corneal stromal fibroblast cells (HCF)	22.4 ± 2.44/22.23 ± 1.99	Control/ KC	A differential expression pattern of autophagy pathway (LC3A, RAB7, LAMP1), oxidative stress, and mTOR were found to be dysregulated.	[66]
RNA-seq	Corneal epithelium	32 ± 6.16/27 ± 8.08	Control (myopia)/ KC	Compared with control (myopia), in KC samples they found downregulated genes: cell–cell communication, cell signaling, WNT, and Notch1 signaling pathways.	[93]
Microarrays	Corneal epithelium and the stroma tissue	75.0 ± 11.2/53.95 ± 12.1	Control/KC	They found several genes known to play a role in KC: including AQP5, S100A8, EGLN3, EGFR1, and SFRP1. These genes are associated in metabolic inflammation and the MAPK pathway.	[94]

All findings presented in this table are based on studies of transcriptomic associations with keratoconus. qPCR: Quantitative Polymerase Chain Reaction. RNA-seq: RNA Sequencing. KC: Keratoconus.

**Table 3:** List of proteomics studies of keratoconus.

Type of Analysis	Methodology Employed	Population Age (Mean)	Sample	Findings	Reference
LC-MS/MS	Tears were collected with Schirmer strip	43.96 ± 6.94/44.88 ± 5.01	Control/KC	They showed upregulation in plastin 3, DNA dC→dU-editing enzyme APOBEC-3A1, tubulin α1C chain, 6-phosphogluconate dehydrogenase, decarboxylating, cofilin 1, annexin A2, and annexin A1. Downregulation was shown in serotransferrin, serum albumin, vitamin D binding protein, α1-acid glycoprotein 1, transthyretin, α2-HS-glycoprotein, hemopexin, angiotensinogen, latexin, heat shock cognate 71-kDa protein, and ρ GDP-dissociation inhibitor 1.	[112]
MALDI-TOF MS	Tears were collected with Schirmer strip	30.8 ± 7.6/31.3 ± 7.8	Control/KC	The MS analysis revealed a difference in the IGKC (Immunoglobulin Kappa Chain) protein, lactoferrin, and zinc-α2-glycoprotein (ZAG).	[113]
PEA	Tears were collected with Schirmer strip	35.5 ± 14.9/36.9 ± 13.5	AC/KC	There were notable differences between AC and KC in MLN and PTH1R, five-fold for IFNG and TPSAB1, and four-fold for IL17F, ITGA6, and ISM1. Additionally, they identified biological processes associated with the regulation of gene expression, cell population proliferation, transcription by RNA polymerase II, ERK1/2 cascade, and immune responses in AC and KC.	[114]
LC-MS/MS	Corneal tissue	62.4 ± 12.4/27.5 ± 8.04	Control/KC	Of 3132 proteins, 205 were modified with proline oxidation and all of the detected collagens fell. The associated biological processes were EIF2 signaling, complement system, regulation of eIF4 and p70S6K signaling, acute-phase response signaling, and mTOR signaling.	[115]

All findings presented in this table are based on studies of proteomic associations with keratoconus. LC-MS/MS: Liquid Chromatography–Tandem Mass Spectrometry. AC: Allergic Conjunctivitis. KC: Keratoconus. PEA: The proximity extension assay. MALDI-TOF MS: Matrix-Assisted Laser Desorption/Ionization Time-Of-Flight Mass Spectrometry.

**Table 4:** List of epigenomics studies of keratoconus.

Type of Analysis	Methodology Employed	Population Age (Mean)	Sample	Findings	Reference
Epigenomic	Corneal epithelium	52 ± 24.92/41.6 ± 7.81	Control/KC	Methylated regions were found in linkage loci (3p14.3, 5q35.2, 13q32.3, 15q24.1, and 20p13) and DNA methylation changes in WNT3 and WNT5A.	[45]
Microarrays	Corneal epithelium and the stroma tissue	75.0 ± 11.2/53.95 ± 12.1	Control/KC	Downregulated miRNAs in KC epithelium or KC stroma were miR-634 and miR-936. In addition, the most upregulated miRNAs were miR-211-3p, miR184, and miR-135b-5p.	[94]
ATAC-seq	Immortalized corneal epithelial and keratocyte cell lines		KC	They reported fine-mapping identifies a subset of 55 highly likely causal variants, 91% of which are non-coding. They identified regions of open chromatin, for example, locus 101 associated with DPF3 gene a transcription regulator, histone acetyl-lysine reader.	[128]

All findings presented in this table are based on studies of epigenomic associations with keratoconus. ATAC-seq: Assay for Transposase-Accessible Chromatin with Sequencing. miRNAs: microRNAs. KC: Keratoconus.

**Table 5:** List of metabolomics studies of keratoconus.

Type of Analysis	Methodology Employed	Population Age (Mean)	Sample	Findings	References
GC/MS		54.5/50.5	Control/KC	They identified 13 metabolites whose levels differentiated between groups of samples. Downregulation of several carboxylic acids, fatty acids, and steroids was observed in KC. These metabolites were associated with lipid metabolism and energy production.	[60]
LC-MS/MS	Tears were collected in capillary glass	38 ± 7.02/29.7 ± 9.27	Control/KC	A total of 296 different metabolites were identified, glycolysis and gluconeogenesis had significant changes, such as 3-phosphoglycerate and 1,3 diphopshateglycerate. As a result, the citric acid cycle (TCA) changes in isocitrate, aconitate, malate, and acetylphosphate were shown.	[147]
LC-Q-ToF/MS	Serum	31.5	Control/KC	Upregulated dehydroepiandrosterone sulfate from the steroidal hormone synthesis pathway and metabolic pathways associated with prostaglandin F2α, prostaglandin A2.	[148]
LC-MS	Epithelialized rabbit corneas		Corneal epithelium of rabbits/WST-D/NIR crosslinking/RF-D/UVA crosslinking/received no further treatment.	The pathways were significantly downregulated in RHOB GTPase cycle, repression of retinoic acid–induced cell differentiation, creatine metabolism, activation of the phototransduction cascade, GP1b-IX-V activation signaling. Another significantly downregulated pathway was the activation of matrix metalloproteinases (MMPs) related to ECM organization, which included TIMP-2 and MMP-2.	

All findings presented in this table are based on studies of proteomic associations with keratoconus. LC-MS/MS: Liquid Chromatography–Tandem Mass Spectrometry. KC: Keratoconus. MALDI-TOF MS: LC-Q-ToF/MS: Liquid Chromatography Quadrupole Time-of-Flight Mass Spectrometry. GC/MS: Gas Chromatography and Mass Spectrometry. WST-D/NIR cross-linking: Wavelength-Specific Transglutaminase-Derived/Near-Infrared Cross-Linking. RF-D/UVA Cross-Linking: Radiofrequency-Driven/Ultraviolet-A Cross-Linking.

## Data Availability

No new data were created or analyzed in this study.

## References

[1] Galvis V., Sherwin T., Tello A., Merayo J., Barrera R., Acera A. (2015). Keratoconus: An inflammatory disorder?. Eye.

[2] Mohammadpour M., Heidari Z., Hashemi H. (2017). Updates on Managements for Keratoconus. J. Curr. Ophthalmol..

[3] Valdez-García J.E., Sepúlveda R., Salazar-Martínez J.J., Lozano-Ramírez J.F. (2014). Prevalence of keratoconus in an adolescent population. Rev. Mex. Oftalmol..

[4] Gomes J.A.P., Rodrigues P.F., Lamazales L.L. (2022). Keratoconus epidemiology: A review. Saudi J. Ophthalmol..

[5] Galvis V., Tello A., Jaramillo J.A., Gutierrez A.J., Rodriguez L., Quintero M.P. (2011). Prevalence of keratoconus patients who consulted with a desire refractive surgery in ophthalmology center reference Bucaramanga, Colombia. Rev. Soc. Colomb. Oftal.

[6] Al Qahtani N.A., Abahussin M.O., Assiri A.A. (2022). Demographic and clinical variations of keratoconus in Saudi population. Saudi J. Ophthalmol..

[7] Marx-Gross S., Fieß A., Münzel T., Wild P.S., Beutel M.E., Schmidtmann I., Lackner K.J., Pfeiffer N., Schuster A.K.-G. (2023). Much higher prevalence of keratoconus than announced results of the Gutenberg Health Study (GHS). Graefes Arch. Clin. Exp. Ophthalmol..

[8] Mejia-Salgado G., Cifuentes-González C., Rojas-Carabali W., Zarate-Pinzón L., Peña-Pulgar L.F., Polania D., Cruz-Reyes D.L., de-la-Torre A. (2023). Colombian Ocular Diseases Epidemiology Study (CODES): Incidence and sociodemographic characterisation of keratoconus between 2015 and 2020. BMJ Open Ophthalmol..

[9] Lasagni Vitar R.M., Bonelli F., Rama P., Ferrari G. (2022). Nutritional and Metabolic Imbalance in Keratoconus. Nutrients.

[10] Alves M., Asbell P., Dogru M., Giannaccare G., Grau A., Gregory D., Kim D.H., Marini M.C., Ngo W., Nowinska A. (2023). TFOS Lifestyle Report: Impact of environmental conditions on the ocular surface. Ocul. Surf..

[11] Peris-Martínez C., Piá-Ludeña J.V., Rog-Revert M.J., Fernández-López E., Domingo J.C. (2023). Antioxidant and Anti-Inflammatory Effects of Oral Supplementation with a Highly-Concentrated Docosahexaenoic Acid (DHA) Triglyceride in Patients with Keratoconus: A Randomized Controlled Preliminary Study. Nutrients.

[12] Gordon-Shaag A., Millodot M., Shneor E. (2012). The Epidemiology and Etiology of Keratoconus. Int. J. Keratoconus Ectatic Corneal Dis..

[13] Santodomingo-Rubido J., Carracedo G., Suzaki A., Villa-Collar C., Vincent S.J., Wolffsohn J.S. (2022). Keratoconus: An updated review. Cont. Lens Anterior Eye.

[14] Lucas S.E.M., Burdon K.P. (2020). Genetic and Environmental Risk Factors for Keratoconus. Annu. Rev. Vis. Sci..

[15] Karczewski K.J., Snyder M.P. (2018). Integrative omics for health and disease. Nat. Rev. Genet..

[16] Sun X., Zhang H., Shan M., Dong Y., Zhang L., Chen L., Wang Y. (2022). Comprehensive Transcriptome Analysis of Patients With Keratoconus Highlights the Regulation of Immune Responses and Inflammatory Processes. Front. Genet..

[17] Christensen N.J., Demharter S., Machado M., Pedersen L., Salvatore M., Stentoft-Hansen V., Iglesias M.T. (2022). Identifying interactions in omics data for clinical biomarker discovery using symbolic regression. Bioinformatics.

[18] Hao X.-D., Chen X.-N., Zhang Y.-Y., Chen P., Wei C., Shi W.-Y., Gao H. (2020). Multi-level consistent changes of the ECM pathway identified in a typical keratoconus twin’s family by multi-omics analysis. Orphanet J. Rare Dis..

[19] Chen X., Liu C., Cui Z., Huang Y., Luo Q., Chen S., Wang X., Hou X., Gong Q., Li Y. (2023). Integrative transcriptomics analysis and experimental validation reveal immunomodulatory patterns in keratoconus. Exp. Eye Res..

[20] Kannan R., Shetty R., Panigrahi T., Koh S.K., Khamar P., Deshpande V., Nuijts R.M.M.A., Gijs M., Nishtala K., Zhou L. (2025). Untargeted Tear Proteomics in a Large South-Asian Cohort Reveals Inflammatory Signaling, ECM Remodeling, and Altered Metabolism in Keratoconus. Investig. Ophthalmol. Vis. Sci..

[21] Volatier T.L.A., Figueiredo F.C., Connon C.J. (2020). Keratoconus at a Molecular Level: A Review. Anat. Rec..

[22] Nowak D.M., Gajecka M. (2011). The genetics of keratoconus. Middle East Afr. J. Ophthalmol..

[23] Lalgudi V.G., Shetty R., Nischal K.K., Ziai S., Koaik M., Sethu S. (2022). Biochemical and molecular alterations and potential clinical applications of biomarkers in keratoconus. Saudi J. Ophthalmol..

[24] Shetty R., Sathyanarayanamoorthy A., Ramachandra R.A., Arora V., Ghosh A., Srivatsa P.R., Pahuja N., Nuijts R.M.M.A., Sinha-Roy A., Mohan R.R. (2015). Attenuation of lysyl oxidase and collagen gene expression in keratoconus patient corneal epithelium corresponds to disease severity. Mol. Vis..

[25] Bisceglia L., Ciaschetti M., De Bonis P., Campo P.A.P., Pizzicoli C., Scala C., Grifa M., Ciavarella P., Delle Noci N., Vaira F. (2005). VSX1 mutational analysis in a series of Italian patients affected by keratoconus: Detection of a novel mutation. Investig. Ophthalmol. Vis. Sci..

[26] Jiang X., Boutin T., Vitart V. (2023). Colocalization of corneal resistance factor GWAS loci with GTEx e/sQTLs highlights plausible candidate causal genes for keratoconus postnatal corneal stroma weakening. Front. Genet..

[27] Kim S.-H., Mok J.-W., Kim H.-S., Joo C.K. (2008). Association of -31T>C and -511 C>T polymorphisms in the interleukin 1 beta (IL1B) promoter in Korean keratoconus patients. Mol. Vis..

[28] Wheeler J., Hauser M.A., Afshari N.A., Allingham R.R., Liu Y. (2012). The Genetics of Keratoconus: A Review. Reprod. Syst. Sex. Disord. Curr. Res..

[29] Tyynismaa H., Sistonen P., Tuupanen S., Tervo T., Dammert A., Latvala T., Alitalo T. (2002). A locus for autosomal dominant keratoconus: Linkage to 16q22.3-q23.1 in Finnish families. Investig. Ophthalmol. Vis. Sci..

[30] Brancati F., Valente E.M., Sarkozy A., Fehèr J., Castori M., Del Duca P., Mingarelli R., Pizzuti A., Dallapiccola B. (2004). A locus for autosomal dominant keratoconus maps to human chromosome 3p14-q13. J. Med. Genet..

[31] Nejabat M., Naghash P., Dastsooz H., Mohammadi S., Alipour M., Fardaei M. (2017). VSX1 and SOD1 Mutation Screening in Patients with Keratoconus in the South of Iran. J. Ophthalmic Vis. Res..

[32] Udar N., Atilano S.R., Brown D.J., Holguin B., Small K., Nesburn A.B., Kenney M.C. (2006). SOD1: A candidate gene for keratoconus. Investig. Ophthalmol. Vis. Sci..

[33] Tanwar M., Kumar M., Nayak B., Pathak D., Sharma N., Titiyal J.S., Dada R. (2010). VSX1 gene analysis in keratoconus. Mol. Vis..

[34] Zhang J., Cai B., Ma L., Qin Y., Li S., Sun C., Liang J., Han Y., Zhuang W. (2023). Clinical and genetic analysis VSX1 variants among families with keratoconus in northwest China. Front. Genet..

[35] Hu X., Zhang B., Li X., Li M., Wang Y., Dan H., Zhou J., Wei Y., Ge K., Li P. (2023). The application and progression of CRISPR/Cas9 technology in ophthalmological diseases. Eye.

[36] Virolainen S.J., VonHandorf A., Viel K.C.M.F., Weirauch M.T., Kottyan L.C. (2023). Gene-environment interactions and their impact on human health. Genes. Immun..

[37] Balasubramanian S.A., Pye D.C., Willcox M.D.P. (2010). Are proteinases the reason for keratoconus?. Curr. Eye Res..

[38] Alkozi H.A., Franco R., Pintor J.J. (2017). Epigenetics in the eye: An overview of the most relevant ocular diseases. Front. Genet..

[39] Upaphong P., Thonusin C., Wanichthanaolan O., Chattipakorn N., Chattipakorn S.C. (2024). Consequences of exposure to particulate matter on the ocular surface: Mechanistic insights from cellular mechanisms to epidemiological findings. Environ. Pollut..

[40] Marsit C.J. (2015). Influence of environmental exposure on human epigenetic regulation. J. Exp. Biol..

[41] Cao Q., Xu W., Chen W., Peng D., Liu Q., Dong J., Reinach P.S., Yan D. (2020). MicroRNA-184 negatively regulates corneal epithelial wound healing via targeting CDC25A, CARM1, and LASP1. Eye Vis..

[42] Breton C.V., Landon R., Kahn L.G., Enlow M.B., Peterson A.K., Bastain T., Braun J., Comstock S.S., Duarte C.S., Hipwell A. (2021). Exploring the evidence for epigenetic regulation of environmental influences on child health across generations. Commun. Biol..

[43] Kather J.N., Friedrich J., Woik N., Sticht C., Gretz N., Hammes H.-P., Kroll J. (2014). Angiopoietin-1 Is Regulated by miR-204 and Contributes to Corneal Neovascularization in KLEIP-Deficient Mice. Investig. Ophthalmol. Vis. Sci..

[44] Liao C.-H., Tseng C.-L., Lin S.-L., Liang C.-L., Juo S.-H.H. (2022). MicroRNA Therapy for Dry Eye Disease. J. Ocul. Pharmacol. Ther..

[45] Kabza M., Karolak J.A., Rydzanicz M., Udziela M., Gasperowicz P., Ploski R., Szaflik J.P., Gajecka M. (2019). Multiple Differentially Methylated Regions Specific to Keratoconus Explain Known Keratoconus Linkage Loci. Investig. Ophthalmol. Vis. Sci..

[46] Kabza M., Karolak J.A., Rydzanicz M., Szcześniak M.W., Nowak D.M., Ginter-Matuszewska B., Polakowski P., Ploski R., Szaflik J.P., Gajecka M. (2017). Collagen synthesis disruption and downregulation of core elements of TGF-β, Hippo, and Wnt pathways in keratoconus corneas. Eur. J. Hum. Genet..

[47] Cuellar-Partida G., Springelkamp H., Lucas S.E.M., Yazar S., Hewitt A.W., Iglesias A.I., Montgomery G.W., Martin N.G., Pennell C.E., van Leeuwen E.M. (2015). WNT10A exonic variant increases the risk of keratoconus by decreasing corneal thickness. Hum. Mol. Genet..

[48] Vazirani J., Basu S. (2013). Keratoconus: Current perspectives. Clin. Ophthalmol..

[49] Sorkhabi R., Ghorbanihaghjo A., Taheri N., Ahoor M.H. (2015). Tear film inflammatory mediators in patients with keratoconus. Int. Ophthalmol..

[50] Nichani P.A.H., Solomon B., Trinh T., Mimouni M., Rootman D., Singal N., Chan C.C. (2023). Investigating the role of inflammation in keratoconus: A retrospective analysis of 551 eyes. Eur. J. Ophthalmol..

[51] Loh I.-P., Sherwin T. (2022). Is Keratoconus an Inflammatory Disease? The Implication of Inflammatory Pathways. Ocul. Immunol. Inflamm..

[52] Khaled M.L., Helwa I., Drewry M., Seremwe M., Estes A., Liu Y. (2017). Molecular and Histopathological Changes Associated with Keratoconus. Biomed. Res. Int..

[53] Teo A.W.J., Mansoor H., Sim N., Lin M.T.-Y., Liu Y.-C. (2022). In Vivo Confocal Microscopy Evaluation in Patients with Keratoconus. J. Clin. Med..

[54] Ionescu C., Corbu C.G., Tanase C., Jonescu-Cuypers C., Nicula C., Dascalescu D., Cristea M., Voinea L.-M. (2016). Inflammatory Biomarkers Profile as Microenvironmental Expression in Keratoconus. Dis. Markers.

[55] Shetty R., Ghosh A., Lim R.R., Subramani M., Mihir K., R R.A., Ranganath A., Nagaraj S., Nuijts R.M.M.A., Beuerman R. (2015). Elevated Expression of Matrix Metalloproteinase-9 and Inflammatory Cytokines in Keratoconus Patients Is Inhibited by Cyclosporine A. Investig. Ophthalmol. Vis. Sci..

[56] Nabil K.M., Elhady G.M., Morsy H. (2019). The Association Between Interleukin 1 Beta Promoter Polymorphisms And Keratoconus Incidence And Severity In An Egyptian Population. Clin. Ophthalmol..

[57] Tseng H.-C., Lee I.-T., Lin C.-C., Chi P.-L., Cheng S.-E., Shih R.-H., Hsiao L.-D., Yang C.-M. (2013). IL-1β promotes corneal epithelial cell migration by increasing MMP-9 expression through NF-κB- and AP-1-dependent pathways. PLoS ONE.

[58] Balmus I.-M., Alexa A.I., Ciuntu R.-E., Danielescu C., Stoica B., Cojocaru S.I., Ciobica A., Cantemir A. (2020). Oxidative stress markers dynamics in keratoconus patients’ tears before and after corneal collagen crosslinking procedure. Exp. Eye Res..

[59] Dammak A., Pastrana C., Martin-Gil A., Carpena-Torres C., Peral Cerda A., Simovart M., Alarma P., Huete-Toral F., Carracedo G. (2023). Oxidative Stress in the Anterior Ocular Diseases: Diagnostic and Treatment. Biomedicines.

[60] Wojakowska A., Pietrowska M., Widlak P., Dobrowolski D., Wylęgała E., Tarnawska D. (2020). Metabolomic signature discriminates normal human cornea from Keratoconus—A pilot GC/MS study. Molecules.

[61] Lema I., Sobrino T., Durán J.A., Brea D., Díez-Feijoo E. (2009). Subclinical keratoconus and inflammatory molecules from tears. Br. J. Ophthalmol..

[62] Atilano S.R., Lee D.H., Fukuhara P.S., Chwa M., Nesburn A.B., Udar N., Kenney M.C. (2019). Corneal Oxidative Damage in Keratoconus Cells due to Decreased Oxidant Elimination from Modified Expression Levels of SOD Enzymes, PRDX6, SCARA3, CPSF3, and FOXM1. J. Ophthalmic Vis. Res..

[63] Biswas S.K. (2016). Does the Interdependence between Oxidative Stress and Inflammation Explain the Antioxidant Paradox?. Oxid. Med. Cell. Longev..

[64] Soiberman U., Foster J.W., Jun A.S., Chakravarti S. (2017). Pathophysiology of Keratoconus: What Do We Know Today. Open Ophthalmol. J..

[65] Mohaghegh S., Kangari H., Masoumi S.J., Bamdad S., Rahmani S., Abdi S., Fazil N., Shahbazi S. (2023). Prevalence and risk factors of keratoconus (including oxidative stress biomarkers) in a cohort study of Shiraz university of medical science employees in Iran. BMC Ophthalmol..

[66] Shetty R., Sharma A., Pahuja N., Chevour P., Padmajan N., Dhamodaran K., Jayadev C., Nuijts R.M.M.A., Ghosh A., Nallathambi J. (2017). Oxidative stress induces dysregulated autophagy in corneal epithelium of keratoconus patients. PLoS ONE.

[67] Matthews F.J., Cook S.D., Majid M.A., Dick A.D., Smith V.A. (2007). Changes in the balance of the tissue inhibitor of matrix metalloproteinases (TIMPs)-1 and -3 may promote keratocyte apoptosis in keratoconus. Exp. Eye Res..

[68] Nowak-Wąs M., Wąs P., Czuba Z., Wojnicz R., Wyględowska-Promieńska D. (2024). Expression of Tissue Inhibitors of Metalloproteinases (TIMP-1, TIMP-2, TIMP-3, TIMP-4) in Blood Serum of Patients with Keratoconus. J. Clin. Med..

[69] Kaldawy R.M., Wagner J., Ching S., Seigel G.M. (2002). Evidence of apoptotic cell death in keratoconus. Cornea.

[70] Chen C., Wang J., Pan D., Wang X., Xu Y., Yan J., Wang L., Yang X., Yang M., Liu G.-P. (2023). Applications of multi-omics analysis in human diseases. MedComm.

[71] Kim D.-H., Kim Y.-S., Son N.-I., Kang C.-K., Kim A.-R. (2017). Recent omics technologies and their emerging applications for personalised medicine. IET Syst. Biol..

[72] Delpierre C., Lefèvre T. (2023). Precision and personalized medicine: What their current definition says and silences about the model of health they promote. Implication for the development of personalized health. Front. Sociol..

[73] Tebani A., Afonso C., Marret S., Bekri S. (2016). Omics-Based Strategies in Precision Medicine: Toward a Paradigm Shift in Inborn Errors of Metabolism Investigations. Int. J. Mol. Sci..

[74] Gasperskaja E., Kučinskas V. (2017). The most common technologies and tools for functional genome analysis. Acta Medica Litu..

[75] Donath X., Saint-Martin C., Dubois-Laforgue D., Rajasingham R., Mifsud F., Ciangura C., Timsit J., Bellanné-Chantelot C. (2019). Next-generation sequencing identifies monogenic diabetes in 16% of patients with late adolescence/adult-onset diabetes selected on a clinical basis: A cross-sectional analysis. BMC Med..

[76] Orsini A., Diquigiovanni C., Bonora E. (2023). Omics Technologies Improving Breast Cancer Research and Diagnostics. Int. J. Mol. Sci..

[77] Mu A., Klare W.P., Baines S.L., Ignatius Pang C.N., Guérillot R., Harbison-Price N., Keller N., Wilksch J., Nhu N.T.K., Phan M.-D. (2023). Integrative omics identifies conserved and pathogen-specific responses of sepsis-causing bacteria. Nat. Commun..

[78] Hamel A.R., Yan W., Rouhana J.M., Monovarfeshani A., Jiang X., Mehta P.A., Advani J., Luo Y., Liang Q., Rajasundaram S. (2024). Integrating genetic regulation and single-cell expression with GWAS prioritizes causal genes and cell types for glaucoma. Nat. Commun..

[79] Emilsson V., Gudmundsson E.F., Jonmundsson T., Jonsson B.G., Twarog M., Gudmundsdottir V., Li Z., Finkel N., Poor S., Liu X. (2022). A proteogenomic signature of age-related macular degeneration in blood. Nat. Commun..

[80] Fineide F.A., Tashbayev B., Elgstøen K.B.P., Sandås E.M., Rootwelt H., Hynne H., Chen X., Ræder S., Vehof J., Dartt D. (2023). Tear and Saliva Metabolomics in Evaporative Dry Eye Disease in Females. Metabolites.

[81] Yehia L., Eng C. (2023). Genetics and genomics in healthcare: The future is now. Singap. Med. J..

[82] Guigo R., de Hoon M. (2018). Recent advances in functional genome analysis. F1000Research.

[83] Molster C.M., Bowman F.L., Bilkey G.A., Cho A.S., Burns B.L., Nowak K.J., Dawkins H.J.S. (2018). The Evolution of Public Health Genomics: Exploring Its Past, Present, and Future. Front. Public Heal..

[84] Hardcastle A.J., Liskova P., Bykhovskaya Y., McComish B.J., Davidson A.E., Inglehearn C.F., Li X., Choquet H., Habeeb M., Lucas S.E.M. (2021). A multi-ethnic genome-wide association study implicates collagen matrix integrity and cell differentiation pathways in keratoconus. Commun. Biol..

[85] Niazi S., Moshirfar M., Alizadeh F., Doroodgar F., Baradaran-Rafii A., Filutowski O., Niazi F., Ambrósio R.J. (2023). Association of 2 Lysyl Oxidase Gene Single Nucleotide Polymorphisms with Keratoconus: A Nationwide Registration Study. Ophthalmol. Sci..

[86] Hosoda Y., Miyake M., Meguro A., Tabara Y., Iwai S., Ueda-Arakawa N., Nakano E., Mori Y., Yoshikawa M., Nakanishi H. (2020). Keratoconus-susceptibility gene identification by corneal thickness genome-wide association study and artificial intelligence IBM Watson. Commun. Biol..

[87] He W., Han X., Ong J.-S., Hewitt A.W., Mackey D.A., Gharahkhani P., MacGregor S. (2022). Association of Novel Loci With Keratoconus Susceptibility in a Multitrait Genome-Wide Association Study of the UK Biobank Database and Canadian Longitudinal Study on Aging. JAMA Ophthalmol..

[88] Postel M.D., Culver J.O., Ricker C., Craig D.W. (2022). Transcriptome analysis provides critical answers to the “variants of uncertain significance” conundrum. Hum. Mutat..

[89] Kolobkov D.S., Sviridova D.A., Abilev S.K., Kuzovlev A.N., Salnikova L.E. (2022). Genes and Diseases: Insights from Transcriptomics Studies. Genes.

[90] Kukurba K.R., Montgomery S.B. (2015). RNA Sequencing and Analysis. Cold Spring Harb. Protoc..

[91] Lupasco T., He Z., Cassagne M., Sagnial T., Brion L., Fournié P., Gain P., Thuret G., Allouche M., Malecaze F. (2022). Corneal epithelium in keratoconus underexpresses active NRF2 and a subset of oxidative stress-related genes. PLoS ONE.

[92] Shinde V., Hu N., Mahale A., Maiti G., Daoud Y., Eberhart C.G., Maktabi A., Jun A.S., Al-Swailem S.A., Chakravarti S. (2020). RNA sequencing of corneas from two keratoconus patient groups identifies potential biomarkers and decreased NRF2-antioxidant responses. Sci. Rep..

[93] You J., Corley S.M., Wen L., Hodge C., Höllhumer R., Madigan M.C., Wilkins M.R., Sutton G. (2018). RNA-Seq analysis and comparison of corneal epithelium in keratoconus and myopia patients. Sci. Rep..

[94] Stachon T., Nastaranpour M., Seitz B., Meese E., Latta L., Taneri S., Ardjomand N., Szentmáry N., Ludwig N. (2022). Altered Regulation of mRNA and miRNA Expression in Epithelial and Stromal Tissue of Keratoconus Corneas. Investig. Ophthalmol. Vis. Sci..

[95] McMonnies C.W. (2015). Inflammation and keratoconus. Optom. Vis. Sci..

[96] Dou S., Wang Q., Zhang B., Wei C., Wang H., Liu T., Duan H., Jiang H., Liu M., Qi X. (2022). Single-cell atlas of keratoconus corneas revealed aberrant transcriptional signatures and implicated mechanical stretch as a trigger for keratoconus pathogenesis. Cell Discov..

[97] Krok M., Wróblewska-Czajka E., Łach-Wojnarowicz O., Bronikowska J., Czuba Z.P., Wylęgała E., Dobrowolski D. (2024). Analysis of Cytokine and Chemokine Level in Tear Film in Keratoconus Patients before and after Corneal Cross-Linking (CXL) Treatment. Int. J. Mol. Sci..

[98] Sharif R., Khaled M.L., McKay T.B., Liu Y., Karamichos D. (2019). Transcriptional profiling of corneal stromal cells derived from patients with keratoconus. Sci. Rep..

[99] Niu X., Xu M., Zhu J., Zhang S., Yang Y. (2023). Identification of the immune-associated characteristics and predictive biomarkers of keratoconus based on single-cell RNA-sequencing and bulk RNA-sequencing. Front. Immunol..

[100] D’Souza S., Nair A.P., Sahu G.R., Vaidya T., Shetty R., Khamar P., Mullick R., Gupta S., Dickman M.M., Nuijts R.M.M.A. (2021). Keratoconus patients exhibit a distinct ocular surface immune cell and inflammatory profile. Sci. Rep..

[101] Wolf J., Boneva S., Schlecht A., Lapp T., Auw-Haedrich C., Lagrèze W., Agostini H., Reinhard T., Schlunck G., Lange C. (2022). The Human Eye Transcriptome Atlas: A searchable comparative transcriptome database for healthy and diseased human eye tissue. Genomics.

[102] Gobena S., Admassu B., Kinde M.Z., Gessese A.T. (2024). Proteomics and Its Current Application in Biomedical Area: Concise Review. Sci. World J..

[103] Gou W., Yue L., Tang X.-Y., Wu Y.-Y., Cai X., Shuai M., Miao Z., Fu Y., Chen H., Jiang Z. (2022). Circulating Proteome and Progression of Type 2 Diabetes. J. Clin. Endocrinol. Metab..

[104] Kline R.A., Lößlein L., Kurian D., Aguilar Martí J., Eaton S.L., Court F.A., Gillingwater T.H., Wishart T.M. (2022). An Optimized Comparative Proteomic Approach as a Tool in Neurodegenerative Disease Research. Cells.

[105] Starodubtseva N.L., Tokareva A.O., Rodionov V.V., Brzhozovskiy A.G., Bugrova A.E., Chagovets V.V., Kometova V.V., Kukaev E.N., Soares N.C., Kovalev G.I. (2023). Integrating Proteomics and Lipidomics for Evaluating the Risk of Breast Cancer Progression: A Pilot Study. Biomedicines.

[106] de Freitas Campos C., Cole N., Van Dyk D., Walsh B.J., Diakos P., Almeida D., Torrecilhas A., Laus J.L., Willcox M.D.P. (2008). Proteomic analysis of dog tears for potential cancer markers. Res. Vet. Sci..

[107] You J., Willcox M.D., Madigan M.C., Wasinger V., Schiller B., Walsh B.J., Graham P.H., Kearsley J.H., Li Y. (2013). Tear Fluid Protein Biomarkers. Adv. Clin. Chem..

[108] Aass C., Norheim I., Eriksen E.F., Thorsby P.M., Pepaj M. (2015). Single unit filter-aided method for fast proteomic analysis of tear fluid. Anal. Biochem..

[109] Azkargorta M., Soria J., Acera A., Iloro I., Elortza F. (2017). Human tear proteomics and peptidomics in ophthalmology: Toward the translation of proteomic biomarkers into clinical practice. J. Proteom..

[110] Ghosh A., Zhou L., Ghosh A., Shetty R., Beuerman R. (2013). Proteomic and gene expression patterns of keratoconus. Indian J. Ophthalmol..

[111] Ghosh R., Gilda J.E., Gomes A.V. (2014). The necessity of and strategies for improving confidence in the accuracy of western blots. Expert Rev. Proteom..

[112] López-López M., Regueiro U., Bravo S.B., Chantada-Vázquez M.D.P., Varela-Fernández R., Ávila-Gómez P., Hervella P., Lema I. (2021). Tear Proteomics in Keratoconus: A Quantitative SWATH-MS Analysis. Investig. Ophthalmol. Vis. Sci..

[113] Lema I., Brea D., Rodríguez-González R., Díez-Feijoo E., Sobrino T. (2010). Proteomic analysis of the tear film in patients with keratoconus. Mol. Vis..

[114] Gijs M., Adelaar T.I., Vergouwen D.P.C., Visser N., Dickman M.M., Ollivier R.C.I., Berendschot T.T.J.M., Nuijts R.M.M.A. (2023). Tear Fluid Inflammatory Proteome Analysis Highlights Similarities Between Keratoconus and Allergic Conjunctivitis. Investig. Ophthalmol. Vis. Sci..

[115] Shinde V., Hu N., Renuse S., Mahale A., Pandey A., Eberhart C., Stone D., Al-Swailem S.A., Maktabi A., Chakravarti S. (2019). Mapping Keratoconus Molecular Substrates by Multiplexed High-Resolution Proteomics of Unpooled Corneas. OMICS.

[116] López-López M., Regueiro U., Bravo S.B., Chantada-Vázquez M.D.P., Pena C., Díez-Feijoo E., Hervella P., Lema I. (2022). Shotgun Proteomics for the Identification and Profiling of the Tear Proteome of Keratoconus Patients. Investig. Ophthalmol. Vis. Sci..

[117] Li X., Chen K., Wang Z., Li J., Wang X., Xie C., Tong J., Shen Y. (2023). The mTOR signalling in corneal diseases: A recent update. Biochem. Pharmacol..

[118] McKay T.B., Priyadarsini S., Rowsey T., Karamichos D. (2021). Arginine Supplementation Promotes Extracellular Matrix and Metabolic Changes in Keratoconus. Cells.

[119] Rozek L.S., Dolinoy D.C., Sartor M.A., Omenn G.S. (2014). Epigenetics: Relevance and implications for public health. Annu. Rev. Public Health.

[120] Zhang L., Lu Q., Chang C. (2020). Epigenetics in Health and Disease. Adv. Exp. Med. Biol..

[121] Alegría-Torres J.A., Baccarelli A., Bollati V. (2011). Epigenetics and lifestyle. Epigenomics.

[122] Hardy T.M., Tollefsbol T.O. (2011). Epigenetic diet: Impact on the epigenome and cancer. Epigenomics.

[123] Lefkowitz R.B., Miller C.M., Martinez-Caballero J.D., Ramos I. (2024). Epigenetic Control of Innate Immunity: Consequences of Acute Respiratory Virus Infection. Viruses.

[124] Kim Y.J., Yeon Y., Lee W.J., Shin Y.U., Cho H., Sung Y.K., Kim D.R., Lim H.W., Kang M.H. (2019). Comparison of microRNA expression in tears of normal subjects and Sjögren syndrome patients. Investig. Ophthalmol. Vis. Sci..

[125] Kowluru R.A. (2023). Cross Talks between Oxidative Stress, Inflammation and Epigenetics in Diabetic Retinopathy. Cells.

[126] Puigoriol-Illamola D., Martínez-Damas M., Griñán-Ferré C., Pallàs M. (2020). Chronic Mild Stress Modified Epigenetic Mechanisms Leading to Accelerated Senescence and Impaired Cognitive Performance in Mice. Int. J. Mol. Sci..

[127] McMonnies C.W. (2014). Epigenetic Mechanisms Might Help Explain Environmental Contributions to the Pathogenesis of Keratoconus. Eye Contact Lens.

[128] Jiang X., Dellepiane N., Pairo-Castineira E., Boutin T., Kumar Y., Bickmore W.A., Vitart V. (2020). Fine-mapping and cell-specific enrichment at corneal resistance factor loci prioritize candidate causal regulatory variants. Commun. Biol..

[129] Drewry M., Helwa I., Allingham R.R., Hauser M.A., Liu Y. (2016). miRNA Profile in Three Different Normal Human Ocular Tissues by miRNA-Seq. Investig. Ophthalmol. Vis. Sci..

[130] An J., Chen X., Chen W., Liang R., Reinach P.S., Yan D., Tu L. (2015). MicroRNA Expression Profile and the Role of miR-204 in Corneal Wound Healing. Investig. Ophthalmol. Vis. Sci..

[131] Stunf Pukl S. (2022). Are miRNAs Dynamic Biomarkers in Keratoconus? A Review of the Literature. Genes.

[132] Kalaimani L., Devarajan B., Subramanian U., Ayyasamy V., Namperumalsamy V.P., Veerappan M., Chidambaranathan G.P. (2020). MicroRNA Profiling of Highly Enriched Human Corneal Epithelial Stem Cells by Small RNA Sequencing. Sci. Rep..

[133] Chen K., Li Y., Zhang X., Ullah R., Tong J., Shen Y. (2022). The role of the PI3K/AKT signalling pathway in the corneal epithelium: Recent updates. Cell Death Dis..

[134] Yang S., Zhang J., Tan Y., Wang Y. (2022). Unraveling the mechanobiology of cornea: From bench side to the clinic. Front. Bioeng. Biotechnol..

[135] Urbich C., Kuehbacher A., Dimmeler S. (2008). Role of microRNAs in vascular diseases, inflammation, and angiogenesis. Cardiovasc. Res..

[136] Agarwal A., Kansal V., Farooqi H., Singh V.K., Prasad R. (2023). Differentially deregulated microRNAs contribute to ultraviolet radiation-induced photocarcinogenesis through immunomodulation: An-analysis of microRNAs expression profiling. bioRxiv.

[137] Sieland J., Niederer D., Engeroff T., Vogt L., Troidl C., Schmitz-Rixen T., Banzer W., Troidl K. (2023). Changes in miRNA expression in patients with peripheral arterial vascular disease during moderate- and vigorous-intensity physical activity. Eur. J. Appl. Physiol..

[138] Burgos K., Malenica I., Metpally R., Courtright A., Rakela B., Beach T., Shill H., Adler C., Sabbagh M., Villa S. (2014). Profiles of extracellular miRNA in cerebrospinal fluid and serum from patients with Alzheimer’s and Parkinson’s diseases correlate with disease status and features of pathology. PLoS ONE.

[139] Ramanjaneya M., Priyanka R., Bensila M., Jerobin J., Pawar K., Sathyapalan T., Abou-Samra A.B., Halabi N.M., Moin A.S.M., Atkin S.L. (2022). MiRNA and associated inflammatory changes from baseline to hypoglycemia in type 2 diabetes. Front. Endocrinol..

[140] Pilson Q., Smith S., Jefferies C.A., Ní Gabhann-Dromgoole J., Murphy C.C. (2020). miR-744-5p contributes to ocular inflammation in patients with primary Sjogrens Syndrome. Sci. Rep..

[141] Zhang Y., Che D., Cao Y., Yue Y., He T., Zhu Y., Zhou J. (2022). MicroRNA Profiling in the Aqueous Humor of Keratoconus Eyes. Transl. Vis. Sci. Technol..

[142] Benavides-Aguilar J.A., Morales-Rodríguez J.I., Ambriz-González H., Ruiz-Manriquez L.M., Banerjee A., Pathak S., Duttaroy A.K., Paul S. (2023). The regulatory role of microRNAs in common eye diseases: A brief review. Front. Genet..

[143] Gonzalez-Covarrubias V., Martínez-Martínez E., Del Bosque-Plata L. (2022). The Potential of Metabolomics in Biomedical Applications. Metabolites.

[144] Letertre M.P.M., Giraudeau P., de Tullio P. (2021). Nuclear Magnetic Resonance Spectroscopy in Clinical Metabolomics and Personalized Medicine: Current Challenges and Perspectives. Front. Mol. Biosci..

[145] Walker D.I., Valvi D., Rothman N., Lan Q., Miller G.W., Jones D.P. (2019). The metabolome: A key measure for exposome research in epidemiology. Curr. Epidemiol. Rep..

[146] Hong M., Tong L., Mehta J.S., Ong H.S. (2023). Impact of Exposomes on Ocular Surface Diseases. Int. J. Mol. Sci..

[147] Karamichos D., Zieske J.D., Sejersen H., Sarker-Nag A., Asara J.M., Hjortdal J. (2015). Tear metabolite changes in keratoconus. Exp. Eye Res..

[148] Daphne Teh A.L., Jayapalan J.J., Loke M.F., Wan Abdul Kadir A.J., Subrayan V. (2021). Identification of potential serum metabolic biomarkers for patient with keratoconus using untargeted metabolomics approach. Exp. Eye Res..

[149] Karolak J.A., Ginter-Matuszewska B., Tomela K., Kabza M., Nowak-Malczewska D.M., Rydzanicz M., Polakowski P., Szaflik J.P., Gajecka M. (2020). Further evaluation of differential expression of keratoconus candidate genes in human corneas. PeerJ.

[150] Andrade F.E.C., Covre J.L., Ramos L., Hazarbassanov R.M., Dos Santos M.S., Campos M., Gomes J.Á.P., Gil C.D. (2018). Evaluation of galectin-1 and galectin-3 as prospective biomarkers in keratoconus. Br. J. Ophthalmol..

[151] Sharif R., Hjortdal J., Sejersen H., Frank G., Karamichos D. (2017). Human in vitro Model Reveals the Effects of Collagen Cross-linking on Keratoconus Pathogenesis. Sci. Rep..

[152] Balogun M.M., Fashola M.B. (2023). Association between keratoconus and allergic conjunctivitis in children attending a Tertiary Hospital in Nigeria. Rom. J. Ophthalmol..

[153] Wang Q., Deng Y., Li S., Du X., Zhao X., Zhang T., Yuan J. (2021). Corneal biomechanical changes in allergic conjunctivitis. Eye Vis..

[154] Yu H., Dou S., Wang H., Sun Y., Qu J., Liu T., Liu X., Wei C., Gao H. (2025). Role of m(6)A methyltransferase METTL3 in keratoconus pathogenesis. Exp. Eye Res..

[155] Neveu V., Nicolas G., Amara A., Salek R.M., Scalbert A. (2023). The human microbial exposome: Expanding the Exposome-Explorer database with gut microbial metabolites. Sci. Rep..

[156] Akkaya S., Ulusoy D.M. (2020). Serum Vitamin D Levels in Patients with Keratoconus. Ocul. Immunol. Inflamm..

[157] Markoulli M., Ahmad S., Arcot J., Arita R., Benitez-Del-Castillo J., Caffery B., Downie L.E., Edwards K., Flanagan J., Labetoulle M. (2023). TFOS Lifestyle: Impact of nutrition on the ocular surface. Ocul. Surf..

[158] Koithan M., Devika J. (2010). New Approaches to Nutritional Therapy. J. Nurse Pract..

[159] Fucito M., Spedicato M., Felletti S., Yu A.C., Busin M., Pasti L., Franchina F.A., Cavazzini A., De Luca C., Catani M. (2024). A Look into Ocular Diseases: The Pivotal Role of Omics Sciences in Ophthalmology Research. ACS Meas. Sci. Au.

[160] Shetty R., D’Souza S., Khamar P., Ghosh A., Nuijts R.M.M.A., Sethu S. (2020). Biochemical Markers and Alterations in Keratoconus. Asia-Pac. J. Ophthalmol..

[161] Kanclerz P., Khoramnia R., Wang X. (2021). Current Developments in Corneal Topography and Tomography. Diagnostics.

[162] Niazi S., Gatzioufas Z., Doroodgar F., Findl O., Baradaran-Rafii A., Liechty J., Moshirfar M. (2024). Keratoconus: Exploring fundamentals and future perspectives—A comprehensive systematic review. Ther. Adv. Ophthalmol..

[163] Castro-Luna G., Pérez-Rueda A. (2020). A predictive model for early diagnosis of keratoconus. BMC Ophthalmol..

